# Agarose Gel-Supported Culture of Cryopreserved Calf Testicular Tissues

**DOI:** 10.3390/vetsci12101005

**Published:** 2025-10-17

**Authors:** Daozhen Jiang, Wenqian Zhu, Rui Yang, Boyang Zhang, Yingshu Pan, Yifei Mao, Yueqi Wang, Yan Zhang, Bo Tang, Xueming Zhang

**Affiliations:** 1 College of Veterinary Medicine, Jilin University, Changchun 130062, China; jiangdz23@mails.jlu.edu.cn (D.J.); zhuwq21@mails.jlu.edu.cn (W.Z.); ruiyang22@mails.jlu.edu.cn (R.Y.); zby23@mails.jlu.edu.cn (B.Z.); maoyf24@mails.jlu.edu.cn (Y.M.); yueqiw22@mails.jlu.edu.cn (Y.W.); z_yan22@mails.jlu.edu.cn (Y.Z.); tang_bo@jlu.edu.cn (B.T.); 2 Jilin Provincial Key Laboratory of Molecular and Chemical Genetic, Second Hospital, Jilin University, Changchun 130062, China; 3 College of Animal Science, Jilin University, Changchun 130062, China; panys@jlu.edu.cn

**Keywords:** agarose gel, cattle, post-cryopreservation, cultivation, testis

## Abstract

Optimizing the cultivation system is crucial for tissue culture. The culture of cryopreserved testicular tissues is of great importance for the germplasm preservation of endangered animals and especially to ensure high-quality and high-output livestock. In this study, we compared two cultivation systems (Agarose-Supported system and Direct Adherent system) by evaluating their effects on tissue morphology, cell proliferation, apoptosis, gene expression, and endocrine function in cryopreserved testicular tissues from 30-day-old calves. The testicular tissues were cultured for 18 and 27 days with three biological replicates per group, aiming to identify which system better supports tissue preservation, cellular viability, and spermatogenic differentiation. This allowed us to clarify how different cultivation systems influence the structural maintenance and developmental potential of immature bovine testicular tissues. Histological and gene expression analyses revealed that the Agarose-Supported system better preserved the seminiferous cord architecture and supported the development of the seminiferous epithelium compared to the Direct Adherent system. The Agarose system significantly reduced the apoptosis and enhanced the expression of some key genes, including spermatogonial stem cell (SSC) markers (*GFRα-1*, *UCHL1*), meiotic marker (*SYCP3*), mature sperm marker (*CRISP1*), and testicular somatic cell markers (*STAR*, *SOX9*, *ACTA2*). The Agarose-Supported system also benefited spermatogenic differentiation and testosterone secretion. These findings demonstrate that the Agarose-Supported system facilitates the in vitro development of spermatogenic cells and Leydig cells in post-cryopreserved immature bovine testicular tissues.

## Simple Summary

The ability to culture testicular tissues in vitro provides an important tool for studying male fertility. In this study, we compared two methods for culturing cryopreserved calf testicular tissues: Agarose-Supported culture and Direct Adherent culture. Our findings showed that agarose gel offered a better microenvironment, maintained the tissue structure, reduced apoptosis, and supported germ cell proliferation and differentiation. It also promoted the expression of spermatogenesis-related genes and regulated testosterone secretion. These results suggest that Agarose-Supported culture is a promising approach for preserving and studying testicular tissue development in large livestock.

## 1. Introduction

In vitro culture of testicular tissues has emerged as a key focus in reproductive biology, with wide applications in studying testicular development, reproductive physiology, and reproductive toxicology [1]. Testicular tissue culture systems preserve the native three-dimensional (3D) architecture of testicular tissues and maintain the interactions between germ and somatic cells by effectively mimicking the in vivo environment of spermatogenesis [2]. Such systems provide a valuable model for investigating cell-to-cell signaling and paracrine regulatory pathways involved in spermatogenesis [3,4]. Notably, using agarose gel to establish a gas–liquid interphase culture system has enabled complete spermatogenesis in mouse testicular tissues cultured in vitro [5,6]. More recently, testicular tissue culture has been used as a model to examine the viability of cryopreserved samples. However, in species with a longer spermatogenic cycle, such as large livestock cattle, challenges including tissue hypoxia and low efficiency of spermatogenesis still remain during in vitro culture [3,7,8].

Recently, long-term culture of post-cryopreserved immature human testicular tissues has been successfully achieved using a Millicell insert system. This system provided an air–liquid interface, enhanced germ cell development, and generated haploid cells [9]. Studies have shown that employing agarose gel as the supporting material can also create an air–liquid interface and support the structural integrity and intercellular signaling of the testicular tissues in vitro [5,10,11,12]. Despite these advances, species-specific differences pose challenges. Rodent-based tissue culture systems cannot be directly applied to cattle, where conventional approaches often result in necrosis and germ cell loss due to poor oxygenation and nutrient diffusion. Up to now, whether agarose gel-supported cultivation can be used for post-cryopreserved bovine testicular tissues is unknown. This study aimed to test the hypothesis that the Agarose-Supported system also provides an optimal environment for the in vitro development of immature bovine testicular tissues. Building on our previous work [13,14,15,16], we compared it with the conventional Direct Adherent system in supporting the growth of cryopreserved calf testicular tissues, with the ultimate goal of establishing a culture system that sustains continuous bovine testicular development after cryopreservation.

## 2. Materials and Methods

### 2.1. Experimental Design

**Figure 1:**
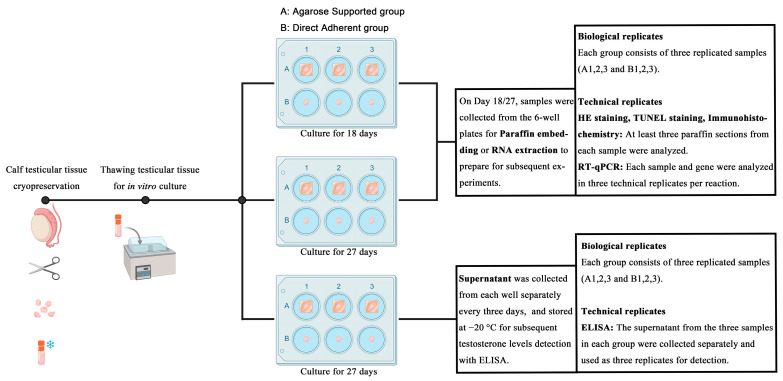
Schematic flowchart of the overall experimental design and workflow. This figure outlines the major steps, including tissue collection, cryopreservation and thawing, in vitro culture under two different systems, sample collection at designated timepoints (D18, D27, or every three days), and subsequent analyses (histology, apoptosis, proliferation, gene expression, and hormone assays).

To facilitate understanding of the experimental workflow, a schematic representation of the overall study design is presented in Figure 1.

### 2.2. Cryopreservation, Thawing, and Culture of Calf Testicular Tissues

The experiments with animals were approved by the Institutional Animal Care and Use Committee of Jilin University (No. SY201903002). Chemicals were purchased from Sigma-Aldrich (St. Louis, MO, USA) and the basic medium was bought from Gibco (Vacaville, CA, USA), unless stated otherwise. The testicular tissues of 30-day-old calves were obtained from Jixing Meat Industry Co., Ltd. (Changchun, China). The tissues were subsequently cryopreserved and finally thawed as described [13,17,18]. Agarose gel-supported culture (Agarose-Supported) and direct tissue adherence culture (Direct Adherent) were employed, and the tissues were cultured for 27 days. The samples were analyzed on Day 18 (D18) and Day 27 (D27), respectively. Briefly, agarose gel (1.5%) was cut into approximately 0.5 cm^3^ blocks and pre-equilibrated by immersion in tissue culture medium (TCM) composed of KnockOut™ DMEM supplemented with 10% knockout serum replacement, 10 ng/mL glial cell-derived neurotrophic factor (GDNF, Peprotech, Cranbury, NJ, USA), 5 IU/L bovine FSH (ShuSheng, Ningbo, China), 1 IU/L chorionic gonadotropin (CG), 10^−6^ M retinol, 34 nM α-tocopherol, 0.05 mg/mL ascorbic acid, 1% nonessential amino acids, 1% L-glutamine, 1% sodium pyruvate, and 1% penicillin-streptomycin, and incubated at 37 °C in a humidified incubator (Thermo3110, Waltham, MA, USA) with 5% CO_2_ for 24 h. The thawed testicular tissues were mechanically minced into approximately 2 mm^3^ fragments. The pre-conditioning medium in the Agarose-Supported group was removed prior to tissue placement. Tissue fragments were then placed either on the surface of agarose gel blocks or directly onto the bottom of the culture wells (one fragment/well), followed by the addition of 1.5 mL fresh TCM to each well. Cultures were maintained at 37 °C in a 5% CO_2_ incubator. Supernatant (1 mL) was collected from each well every three days (i.e., d 3, 6, 9, 12, 15, 18, 21, 24, 27), centrifuged at 2500 rpm for 20 min, and stored at −20 °C for subsequent detection of the testosterone levels with ELISA.

### 2.3. Histological Analysis

The tissues were fixed overnight in 4% paraformaldehyde. Paraffin sections of the testicular tissues were deparaffinized in xylene I and II (15 min each), rehydrated through a graded ethanol series and rinsed in tap water. Hematoxylin-eosin (HE) staining was used for histological analysis. The sections were stained with hematoxylin for 10 min, and incubated with 1% acid alcohol for 10 s. After eosin staining for 1 min, the sections were dehydrated with graded ethanol, cleared in xylene, and mounted with neutral resin. Finally, the cell morphology, seminiferous cord diameter, and structural integrity were observed under a microscope (Nikon TE2000-U, Tokyo, Japan). To compare the diameter and structural integrity of the seminiferous cords, three samples from each group were used, 3–4 representative images were selected, and at least 8–10 cords were measured and analyzed with the image analysis software of the microscope.

### 2.4. Quantitative Real-Time Polymerase Chain Reaction (qRT-PCR)

The cultured testicular tissues were collected on days 18 and 27. After mechanical homogenization using a tissue grinder, the total RNA was extracted using RNAiso Plus (Takara, Kyoto Prefecture, Japan), and reverse transcription was performed using a commercial kit (TransGen, Beijing, China) following the manufacturer’s instructions. The qRT-PCR was conducted to assess the expressions of spermatogonial stem cell (SSC) marker genes (*GFRA-1*, *UCHL1*), SSC differentiation marker *C-KIT*, meiotic marker *SYCP3*, spermiogenesis marker *CRISP1*, Sertoli cell marker *SOX9*, peritubular myoid cell (PMC) marker *ACTA2*, and Leydig cell marker *STAR*. The working concentration of primers was 0.25 μM. The qRT-PCR program was as follows: initial denaturation at 95 °C for 10 min, followed by 45 cycles at 95 °C for 15 s and 60 °C for 60 s. The melting curve analysis was performed by increasing the temperature from 60 °C to 95 °C at a rate of 1.6 °C/s, with signal acquisition every second, and finally cooling at 4 °C. The primers are listed in Table 1. Each group included three biological replicates. GAPDH was used as the internal reference gene, and the relative gene expression was calculated using the 2^−ΔΔCt^ method.

### 2.5. Immunohistochemical Staining

Paraffin sections of the cultured testicular tissues were deparaffinized, rehydrated, and washed with phosphate-buffered saline (PBS). Endogenous peroxidase was blocked with 3% H_2_O_2_ at room temperature (RT) for 15 min. Antigen retrieval was performed by heating the sections in citrate buffer until boiling, cooling to 72 °C, and repeating the cycle twice. After cooling to RT, the sections were blocked with 5% bovine serum albumin (BSA) at 37 °C for 30 min. Primary antibodies/BSA (rabbit anti-Ki67, 1:200 (Affinity, San Francisco, CA, USA); rabbit anti-SYCP3 (Genetex, Shenzhen, China), 1:400; 5% BSA, negative control) were applied and incubated overnight at 4 °C. After PBS washes, the sections were incubated with biotinylated goat anti-rabbit IgG at 37 °C for 30 min, followed by incubation with Streptavidin-Biotin Complex reagent. The DAB (3,3′-Diaminobenzidine) staining was used for color development. The sections were washed with tap water and observed under a light microscope to determine the expression levels of Ki67/SYCP3.

### 2.6. TUNEL Staining

Cell apoptosis in the testicular tissues was evaluated using TUNEL Assay Kit (TransGen Biotech, Beijing, China) as described [13]. Briefly, paraffin sections were deparaffinized, rehydrated, and treated with proteinase K at RT for 20 min. After PBS washes, sections were incubated with TUNEL reaction mixture in a humidified incubator (Thermo3110, Waltham, MA, USA) at 37 °C for 1 h. Positive controls were pre-treated with DNase I. Negative controls were not incubated with the labeling solution. The apoptotic cells were observed under a Nikon 80i fluorescence microscope. The apoptotic rate (%) was calculated as a ratio of the TUNEL positive cell number to the total cell number per visual field.

### 2.7. Testosterone Detection

Testosterone levels in the culture supernatants were measured using a competitive Enzyme-Linked Immunosorbent Assay (ELISA) kit (Nanjing Jiancheng, Nanjing, China) according to the manufacturer’s instructions. Briefly, standard solutions were serially diluted to establish a standard curve. For each well, 50 μL sample and 50 μL biotin-labeled antigen were added and the mixture were incubated at 37 °C for 30 min. After washing with PBS-T, 50 μL Streptavidin-Horseradish Peroxidase was added and the samples were incubated for another 30 min at 37 °C. Following a second wash, 100 μL of substrate solution was added and the samples were incubated in the dark for 10 min. The reaction was ended by adding of 50 μL stop solution. Finally, the OD values of the samples were measured by microplate reader (DeTie HBS-1096A, Nanjing, China) at 450 nm, and testosterone concentrations were calculated from the standard curve.

### 2.8. Statistical Analysis

Statistical analyses were performed using a two-way analysis of variance (2-way ANOVA) to assess the effects of the culture systems and culture time on the seminiferous cord diameter and structural integrity. Tukey test was used as a post hoc analysis to pinpoint which specific groups showed differences. Prior to conducting the 2-way ANOVA, the data were tested for normality using the Shapiro–Wilk test. Apoptosis, proliferation, and testosterone level assays were performed using the same methods as described above. Differences in gene expression were assessed using Independent Two-sample T-tests, with Welch’s correction applied when the assumption of equal variances was violated, and normality was tested beforehand using the Shapiro–Wilk test. Values were considered significantly different when the *p*-value was < 0.05 (* *p <* 0.05, ** *p <* 0.01, **** *p <* 0.0001). For morphological, apoptosis, proliferation, and differentiation analysis, three samples were replicated for each group, and 3–4 images from each sample and 8–10 cords from each image were selected randomly for statistical evaluation. For RT-qPCR, the test of each group was performed with three biological replicates, and each biological replicate was analyzed with three technical replicates. For ELISA, the supernatant from three biological replicates were collected separately and used as three replicates for detection. Data were presented as mean ± SEM.

## 3. Results

### 3.1. Morphological Changes in Calf Testicular Tissues

**Figure 2:**
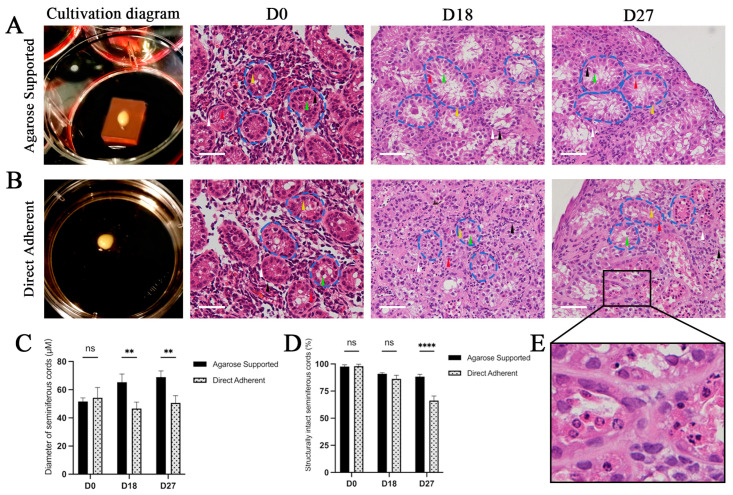
Culture of calf testicular tissues and the histological analysis. (**A**) Testicular tissues cultured with agarose gel (Agarose-Supported), stained with hematoxylin-eosin (HE) at D0, D18, and D27 (Day 0, Day 18, Day 27). (**B**) Testicular tissues cultured by direct tissue adherence (Direct Adherent), stained with HE at D0, D18, and D27. Blue dashed lines: seminiferous cords, green triangles: gonocytes, yellow triangles: spermatogonial stem cells, red triangles: Sertoli cells, black triangles: peritubular myoid cells, white triangles: basement membrane. Scale bar = 50 μm. (**C**) Diameter of seminiferous cords at D0, D18, and D27 in two groups. (**D**) Percentage of structurally intact seminiferous cords at D0, D18, and D27 in two groups. (** *p* < 0.01, **** *p* < 0.0001, ns = no statistical difference between groups). (**E**) The details of the selected area in panel B (D27) are shown at higher magnification to reveal nuclear pyknosis, fragmentation, and basement membrane thickening.

The frozen–thawed calf testicular tissues were cultured using two methods (Figure 2A,B). HE staining revealed that the frozen–thawed tissues retained intact structures, with a small seminiferous cord diameter, 1–2 layers of germinal epithelium, large intercord spaces, and loose interstitial areas rich in capillaries. The gonocytes, immature Sertoli cells (SCs), and peritubular myoid cells (PMCs) were discerned (D0 in Figure 2A,B). During the cultivation, the seminiferous epithelium in the Agarose group remained orderly. The SCs displayed irregular pyramidal shapes extending toward the center of the seminiferous cords, and the gonocytes were embedded between the lateral and apical surfaces of SCs. The PMCs were still closely aligned along the basement membrane, and the intercord spaces decreased with enriched interstitial components (Figure 2A). The diameter of seminiferous cords in the Agarose-Supported group was significantly larger than that in the Direct Adherent group at both D18 and D27 (Figure 2C).

In contrast, structural damage to the seminiferous epithelium was observed in the Direct Adherent group on D27; the proportion of structurally intact seminiferous cords was significantly lower than that in Agarose group (Figure 2D). Some testicular cells exhibited nuclear pyknosis and fragmentation, and thickening of the basement membrane was also observed (Figure 2E). The overall architecture appeared to be collapsed, with fragmented seminiferous cords and disorganized germline layers (Figure 2B). These features are indicative of degenerative changes and may reflect insufficient support for structural maintenance in Direct Adherent group.

### 3.2. Agarose-Supported Culture Decreases Apoptosis and Increases Proliferation

**Figure 3:**
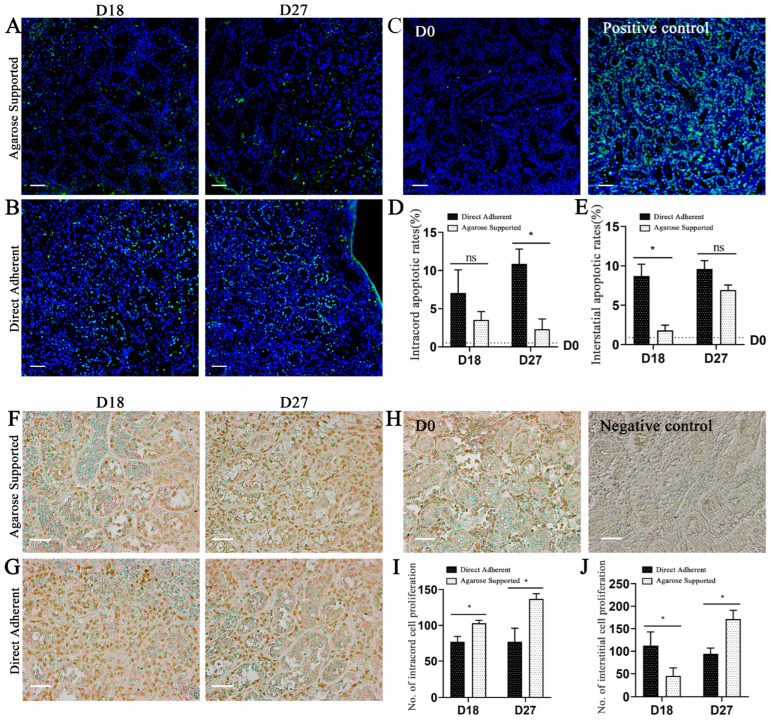
The cell apoptosis and proliferation in the cultured testicular tissues. (**A**,**B**) TUNEL staining of the testicular tissues cultured under Agarose-Supported (**A**) and Direct Adherent (**B**) conditions on D18 and D27, blue indicates nuclei stained with 4’,6-diamidino-2-phenylindole (DAPI), green indicates apoptotic cells. (**C**) TUNEL staining of the testicular tissues at D0 and the positive control. (**D**,**E**) Apoptotic rates in the seminiferous cords and the testicular interstitium, respectively. (**F**,**G**) Ki67 immunohistochemical staining of the testicular tissues cultured under Agarose-Supported (**F**) and Direct Adherent (**G**) conditions on D18 and D27, brown indicates positive expression of the target protein in cells. (**H**) Ki67 staining of the testicular tissues at D0 and the negative control. (**I**,**J**) Statistics of the proliferating cells in the seminiferous cords and the interstitium, respectively. Scale bar = 50 μm. * *p* < 0.05 was considered statistically significant.

The apoptotic analysis revealed that the cryopreserved calf testicular tissues prior to culture exhibited minimal apoptosis (Figure 3C). In contrast, the apoptosis increased following culture (Figure 3A,B). On D18, the interstitial apoptotic rate in the Direct Adherent group was significantly higher than that in the Agarose-Supported group (*p* < 0.05), whereas no significant difference was observed within the seminiferous cords between the two groups (Figure 3D,E). On D27, the number of apoptotic cells within the seminiferous cords in the Direct Adherent group increased remarkably (*p* < 0.05), and the interstitial apoptosis also showed an increasing trend in this group (Figure 3D,E). Immunostaining for the proliferation marker Ki67 indicated varying levels of proliferation in both somatic and germ cells under two conditions (Figure 3F–J). On D18, Agarose culture increased intracord cell proliferation but decreased it in the interstitium. However, by D27, the number of Ki67^+^ cells within the seminiferous cords and interstitial regions both increased significantly in the Agarose group (*p* < 0.05, Figure 3I,J).

### 3.3. Gene Expressions in the Testicular Tissues Cultured with Two Methods

**Figure 4:**
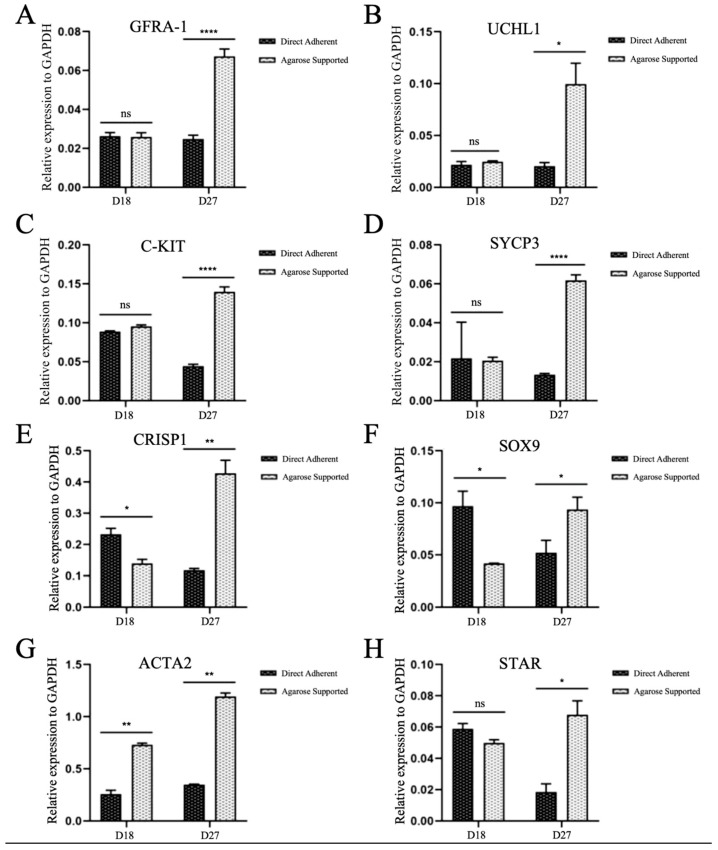
Gene expressions in the cultured calf testicular tissues. The relative mRNA expression of *GFRA-1* (**A**), *UCHL1* (**B**), *C-KIT* (**C**), *SYCP3* (**D**), *CRISP1* (**E**), *SOX9* (**F**), *ACTA2* (**G**), and *STAR* (**H**) were analyzed by qRT-PCR. (* *p* < 0.05, ** *p* < 0.01, **** *p* < 0.0001).

On D18, no statistical differences were detected in the expressions of *GFRA-1*, *UCHL1*, *C-KIT*, *SYCP3*, and *STAR* between the two groups (Figure 4A–D,H; *p* > 0.05); however, the mRNA levels of *CRISP1* and *SOX9* significantly increased in the Direct Adherent group (Figure 4E,F; *p* < 0.05), but the expression of *ACTA2* showed the opposite trend (Figure 4G; *p* < 0.01). With prolonged culture duration (D27), the expression levels of all these genes in the Agarose group exhibited a remarkable elevation (*p* < 0.05, 0.01 or 0.0001, Figure 4A–H).

### 3.4. Effect of Two Culture Methods on Testicular Cell Differentiation

**Figure 5:**
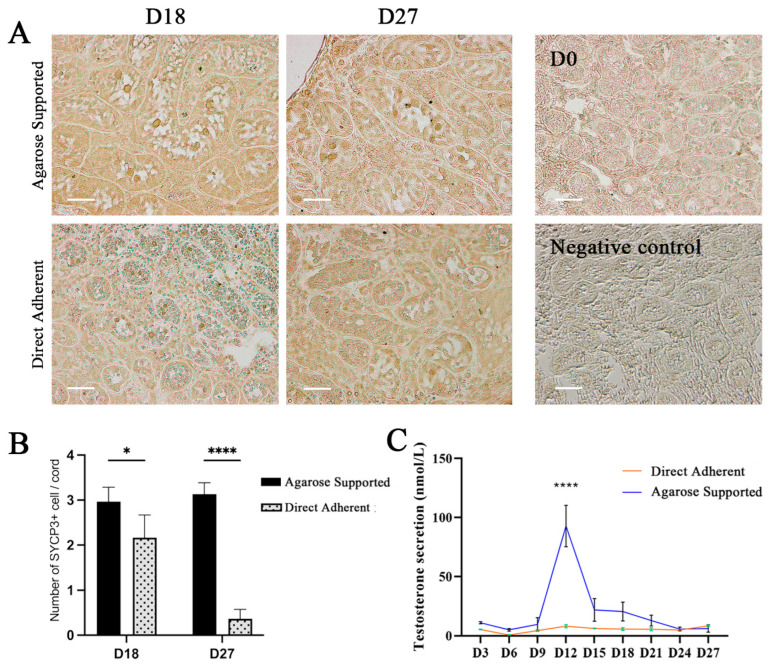
Effect of different culture methods on spermatogenic cell differentiation and testosterone secretion. (**A**) SYCP3 immunostaining was performed on D18 and D27, Scale bar = 50 μm. (**B**) The average number of SYCP3-positive cells in each seminiferous cord. (**C**) Detection of testosterone secretion by ELISA. (* *p* < 0.05, **** *p* < 0.0001).

Immunohistochemical staining for SYCP3, a marker of meiotic spermatogenic cells, revealed the presence of differentiated spermatogenic cells in both two groups on D18. These SYCP3^+^ cells appeared to be sparsely distributed and mostly confined to the seminiferous cords displaying relatively preserved architecture, suggesting limited but ongoing meiotic activity under both culture conditions (Figure 5A). Although a small number of SYCP3^+^ cells were observed in the Adherent group on D18, their quantity was significantly lower compared to the Agarose group. This difference became more evident on D27, with nearly no SYCP3^+^ cells detectable in the Adherent group (Figure 5B).

The ELISA results showed that on D12, testosterone secretion in the Agarose group peaked and was significantly higher (*p* < 0.0001) than that in the Adherent group. At other timepoints, no significant differences was observed between the two groups (Figure 5C). Despite the subsequent decline after D12, testosterone levels in the Agarose group remained relatively stable, while those in the Adherent group showed a flatter, less dynamic profile across the culture period.

## 4. Discussion

In this study, cryopreserved and thawed calf testicular tissues were cultured using two different methods. Both systems initially supported cell proliferation, but significant differences were observed in tissue morphology, apoptosis, and proliferation over time. Testicular tissues cultured with the Agarose system better preserved the structural integrity of the seminiferous cords and demonstrated more robust proliferation and differentiation of various testicular cell types compared to the Adherent system. The Agarose system maintained the 3D architecture and cell–cell interactions crucial for germ cell maintenance, which likely contributed to the sustained proliferative activity of intraseminiferous cord cells. In contrast, the Adherent system led to higher apoptotic rates in the interstitial cells on D18 and intraseminiferous cord cells on D27. This suggests that Agarose system may better support interstitial cell survival by mimicking the native extracellular matrix, facilitating nutrient and oxygen exchange [19,20]. Moreover, apoptotic cells were unevenly distributed within the tissues, with increased apoptosis observed in the central regions compared to the peripheral areas. This shift in apoptotic localization indicates that prolonged culture in the Adherent system compromises intracord cell viability, potentially due to inadequate structural support or suboptimal nutrient/gas exchange within the seminiferous cord microenvironment. Such effects may be linked to the insufficient re-establishment of microvascular blood supply following cryopreservation and thawing in the Direct Adherent culture system. Previous studies have shown that microvascular blood supply can be restored in cryopreserved calf testicular tissues after xenotransplantation [14], suggesting that the Agarose system may create a similar environment to that in xenotransplantation experiments. Recently, Tang et al. demonstrated successful gonocyte dissociation and culture from frozen–thawed neonatal bovine testicular tissue, further forming testicular organoids [21,22], although the microcirculation within these organoids remains unknown.

Regarding gene expression, we observed significant differences between the Agarose and Adherent systems. The upregulation of *SOX9* in the Agarose group indicates better maintenance of Sertoli cell function, which is essential for supporting germ cell survival and proliferation [23], and this was reflected in the preservation of the seminiferous cord integrity and a reduction in cell apoptosis. In contrast, the higher expression of *CRISP1* in the Adherent system suggests more advanced germ cell differentiation, particularly in the post-meiotic stages, but this may also be associated with higher apoptosis due to the lack of structural support and limited cell interactions. The agarose scaffold, with its 3D architecture, appears to create a more favorable environment for both SSCs and differentiated germ cells, as evidenced by the upregulation of *GFRA-1*, *UCHL1*, and *C-KIT*. These data suggest that the Agarose system not only maintains SSCs but also promotes their differentiation into more advanced spermatogenic cells while minimizing apoptosis. Additionally, the increased expression of *ACTA2* in the Agarose group points to the activation of peritubular myoid cells (PMCs), contributing to the structural integrity of seminiferous cords and potentially reducing cellular stress [24], which further enhances cell survival. In addition, activated PMCs may also regulate the expression of testosterone levels by affecting Leydig cells [25]. The upregulation of *STAR* in the Agarose group suggests enhanced Leydig cell activity, which may support germ cell survival by promoting testosterone production [26].

As a critical hormone involved in spermatogenesis, testosterone is primarily synthesized and secreted by interstitial Leydig cells through a series of enzymatic reactions. In the in vitro cultured testicular tissues, hormone production occurs independently of the hypothalamic–pituitary–gonadal axis. In our Agarose group, testosterone concentration peaked early and subsequently declined, likely due to the depletion of hormonal reserves or their endogenous utilization by the cultured tissues. This peak suggests that the Agarose system may better preserve or stimulate Leydig cell function during the early stages of in vitro culture. This finding aligns with previous reports on human testicular tissues [27], where similar patterns of testosterone production were observed in culture systems. The in vivo testosterone secretion in Leydig cells is regulated by the gonadotropin luteinizing hormone (LH). LH stimulates the development of the testicular interstitial Leydig cells; therefore, it is also named as an interstitial cell stimulating hormone. In our Agarose system, LH was excluded in the culture medium since we intended to simplify the medium composition. To improve the current Agarose system in future, LH should be included in the medium formula and its effects on the in vitro development of immature bovine testicular tissues need to be investigated.

## 5. Conclusions

In conclusion, the Agarose-Supported culture better preserved the tissue structure, enhanced the germ cell proliferation, decreased cell apoptosis, and facilitated the development and differentiation of the seminiferous epithelium and the interstitial Leydig cells. The present study provides insights into the feasibility and potential application of testicular organ culture technologies in large domestic animals.

## Figures and Tables

**Table 1:** The primers used in the experiments.

**Gene**	**Forward Primer**	**Reverse Primer**	**Gene ID**
*GAPDH*	CGGCACAGTCAAGGCAGAGAAC	GCACCAGCATCACCCCACTTG	281181
*CRISP1*	ACAGAACTGGAGGCTGTCCAA	ATGTTGCTGGCTGGTGGAGA	616774
*SOX9*	AGGAGAGCGAGGAGGACAAGTTC	ACCAGCGTCCAGTCGTAGCC	100336535
*ACTA2*	GATGGTGGGAATGGGACAGAAAGAC	GGTGATGATGCCGTGCTCTATCG	515610
*STAR*	AAGACCCTCTCTACAGCGACCAAG	GGATCACTTTACTCAGCACCTCGTC	281507
*GFRA-1*	TGGCCCTGCTTGTTTTCCTCT	ACAGGTATGCACGCTTGTGT	534801
*UCHL1*	GATGTTCTGGGACTGGAGGAGGAG	ATGATGGAACCGAGATGCTGCTTC	514394
*C-KIT*	TGTCTGCACTGCTCAGCGAATC	TTGATGGCTGCCCGCACTTTC	280832
*SYCP3*	CCGGGAAGTTGGCAAAACCA	GGCATCCTCCTCTGAACCACT	615896

## Data Availability

The original contributions presented in this study are included in the article. Further inquiries can be directed to the corresponding author.
